# Effects of Dietary Protein Levels on Sheep Gut Metabolite Profiles during the Lactating Stage

**DOI:** 10.3390/ani14010121

**Published:** 2023-12-29

**Authors:** Sikandar Ali, Xiaojun Ni, Muhammad Khan, Xiaoqi Zhao, Hongyuan Yang, Baiji Danzeng, Imtiaz Hussain Raja, Guobo Quan

**Affiliations:** 1 Yunnan Animal Science and Veterinary Institute, Jindian, Panlong District, Kunming 650225, China; sikandar.ptbav@gmail.com (S.A.); xiao666jun@163.com (X.N.); khanbwn011@gmail.com (M.K.); zhao20220311@163.com (X.Z.); yang20211101@163.com (H.Y.); baiji20190101@163.com (B.D.); 2 Zhejiang Vegamax Biotechnology Co., Ltd., Huzhou 313300, China; 3 Yunnan Provincial Animal Genetic Resource Conservation and Germplasm Innovation Engineering Research Center, Jindian, Panlong District, Kunming 650225, China; 4 Department of Animal Nutrition, University of Veterinary and Animal Sciences, Lahore 54000, Pakistan; 5 Department of Animal Nutrition, Faculty of Animal Production & Technology, Cholistan University of Veterinary and Animal Sciences, Bahawalpur 63100, Pakistan; imtiazhussain@cuvas.edu.pk

**Keywords:** sheep, gut, metabolites, metabolome, lactation

## Abstract

**Simple Summary:**

Researchers and breeders are consistently exploring better strategies to improve milk, meat, and wool production in different sheep breeds. Conventionally, sheep are grazed on unbalanced pastures that vary in their composition, which results in lower growth performance and heavy parasitic infections. The ongoing climatic changes also have a drastic impact on grazing areas, leading to severe pasture scarcity across the world. Typically, barn-fed sheep rely on hay with a small amount of grain feeding. Right now, sheep production is pacing toward indoor rearing, which demands a highly precise feeding regimen. Therefore, determining the precise protein levels in the nutrition of indoor sheep production is more critical. The current experiment was planned to evaluate the changes in the rumen metabolite profile of lactating ewes fed different dietary protein levels. Feeding sheep at higher protein levels significantly altered the metabolite profile. These metabolic findings provide insights into potentiated biomarker changes in the metabolism influenced by dietary protein levels.

**Abstract:**

Diet-associated characteristics such as dietary protein levels can modulate the gut’s primary or secondary metabolites, leading to effects on the productive performance and overall health of animals. Whereas fecal metabolite changes are closely associated with gut metabolome, this study aimed to see changes in the rumen metabolite profile of lactating ewes fed different dietary protein levels. For this, eighteen lactating ewes (approximately 2 years old, averaging 38.52 ± 1.57 kg in their initial body weight) were divided into three groups (*n* = 6 ewes/group) by following the complete randomized design, and each group was assigned to one of three low-protein (D_I), medium-protein (D_m), and high-protein (D_h) diets containing 8.58%, 10.34%, and 13.93% crude protein contents on a dry basis, respectively. The fecal samples were subjected to untargeted metabolomics using ultra-performance liquid chromatography (UPLC). The metabolomes of the sheep fed to the high-protein-diet group were distinguished as per principal-component analysis from the medium- and low-protein diets. Fecal metabolite concentrations as well as their patterns were changed by feeding different dietary protein levels. The discriminating metabolites between groups of nursing sheep fed different protein levels were identified using partial least-squares discriminant analysis. The pathway enrichment revealed that dietary protein levels mainly influenced the metabolism-associated pathways (*n* = 63 and 39 in positive as well as negative ionic modes, respectively) followed by protein (*n* = 15 and 8 in positive as well as negative ionic modes, respectively) and amino-acid (*n* = 14 and 7 in positive as well as negative ionic modes, respectively) synthesis. Multivariate and univariate analyses showed comparative changes in the fecal concentrations of metabolites in both positive and negative ionic modes. Major changes were observed in protein metabolism, organic-acid biosynthesis, and fatty-acid oxidation. Pairwise analysis and PCA reveal a higher degree of aggregation within the D-h group than all other pairs. In both the PCA and PLS-DA plots, the comparative separation among the D_h/D_m, D_h/D_I, and D_m/D_I groups was superior in positive as well as negative ionic modes, which indicated that sheep fed higher protein levels had alterations in the levels of the metabolites. These metabolic findings provide insights into potentiated biomarker changes in the metabolism influenced by dietary protein levels. The target identification may further increase our knowledge of sheep gut metabolome, particularly regarding how dietary protein levels influence the molecular mechanisms of nutritional metabolism, growth performance, and milk synthesis of sheep.

## 1. Introduction

Ruminants are well-known foregut fermenters as their rumen hosts a diverse community of microbes that play a fermentation role [1]. Like other ruminants, the sheep’s digestive system makes it efficient to utilize plant-based materials, including protein sources, through a series of fermentation processes. Briefly, rumen microbiota produces certain types of enzymes that are involved in dietary protein degradation into smaller peptides and amino acids [2]. The non-protein nitrogenous part of the dietary protein such as ammonia and urea are utilized by these microorganisms for their growth, resulting in microbial protein contribution to overall protein nutrition [3]. Moreover, protein is a costly nutrient, and dietary concentration can have a direct influence on ruminants, particularly sheep performance as well as economic returns.

Changes in dietary constituents like protein contents can alter the gut microbial community and subsequently impact the metabolites produced by gut bacteria [4]. Studies showed that dietary changes regarding protein quality and quantity have an impact on the rumen ecology and ruminal metabolites of dairy cattle and sheep [4,5,6]. The dietary protein supplies essential amino acids for the growth of fiber-digesting bacteria, which results in changes in metabolite profiling [7]. On the other hand, diets with imbalanced or excessive protein levels may lead to the proliferation of undesirable microbial species, potentially affecting the fermentation process negatively [8]. Therefore, adequate dietary protein is essential for promoting growth and weight gain in ruminants. The dietary proteins supply essential amino acids for building the tissues leading to better growth rates and overall development [9]. For lactating animals, dietary protein supports milk synthesis and improves milk yield, benefiting both the animal and dairy operations [10].

Understanding how varying dietary protein levels can influence the metabolomics of ruminant species is most important to optimize feeding for better production performance, health, and animal welfare. The metabolome is a set of metabolites that are found in biological samples like blood, urine, or tissue of animals [11]. The current advanced techniques can identify the metabolites of rumen microbiota, and it has been reported that changes in some metabolites may affect microbiota alterations and host activity. Currently, metabolomics using mass spectrometry (MS) is a promising tool for the life science and biotechnology fields [12,13]. Approximately 55–60% of rumen fluid metabolites are associated with rumen microbiota [14]. Dietary protein optimization based on metabolomic insights can lead to precise feeding for sustainable livestock production as varying dietary protein levels can lead to changes in metabolite profiles and metabolic pathways. It is established that dietary protein levels have an influence on amino-acid metabolism leading to changes in the concentrations of blood-circulating amino acids and related metabolic pathways in cattle [15]. Keeping in mind the intertwined metabolism of protein with energy, we assumed that changes in dietary protein contents may cause alterations in the concentrations of those metabolites that are involved in energy production, storage, and utilization. Higher concentrations of ketone bodies were detected in the blood of those cattle that were fed on protein-deficient diets, suggesting the breakdown of body proteins [16]. The health and growth of ruminants depend heavily on the production of metabolites in the rumen, a comprehensive analysis of rumen fluid provides insight into the interaction between the diet fed to ruminants and the rumen [14]. The interaction between microbiota and metabolites facilitates the interpretation of microbiota classification and functional properties of some metabolites. In summary, microbiome and metabolomic studies have been widely used in animal nutrition, but there is a lack of reports on the interaction between rumen microbiota and metabolites in lactating Yunnan semi-fine-wool ewes using different dietary protein levels. Yunnan semi-fine-wool sheep are contributing to the income of local farmers in Yunnan province by providing milk, meat, and high-quality wool [17]. Moreover, they are disease-resistant, have excellent adaptation to harsh environmental conditions, and have the ability to thrive on low-quality forage [18]. In this study, the rumen microbiota and metabolites of lactating ewes were analyzed by gene sequencing and LC–MS techniques to understand the effects of different dietary protein levels.

## 2. Materials and Methods

### 2.1. Ethical Approval, Animals, Research Design, and Animal Husbandry

The current experiment was in accordance with the ethical committee of Yunnan Animal Science and Veterinary Institute (201911004), and protocols were in line with the guidelines of the field trials detailed by the State Science and Technology Commission of the People’s Republic of China (Order-No. 2; 1988) and the Standing Committee of Yunnan Provincial People’s Congress, China (No. 10; 2007). The experimental site was Kunming Yixingheng Animal Husbandry Technology Co., Ltd., Kunming, China (26°22′ N; 103°40′ E). In a completely randomized design, eighteen lactating ewes (approximately 2 years old, averaging 38.52 ± 1.57 kg in their initial body weight) were randomized into three groups (*n* = 6 ewes/group), and each group was assigned to one of three diets with varying protein levels. The diets were low protein (D_I), medium protein (D_m), and high protein (D_h), containing 8.58%, 10.34%, and 13.93% crude protein contents, respectively, on a dry basis. The feed formulations and their chemical compositions on a dry basis are detailed in Table 1 and Table 2, respectively. The animal husbandry practices, strategic feeding, and fecal sample collection timing, protocols, and storage were detailed in our previous publications [17,18]. Briefly, On the 136th day of pregnancy, the individually housed ewes were fed on dietary treatments with twice-a-day feeding frequency (8:30 a.m. and 4:00 p.m. feeding times). Corn silage was fed other than these times, while access to fresh water was given on a choice basis during the whole experimental period. On the 91st postpartum day, each ewe was subjected to fecal sampling (approximately 10 g) from the terminal part of the rectum, and the collected samples were immediately stored at −80 °C in labeled 10 mL sterile freezing tubes for metabolomic analysis.

### 2.2. Metabolite Extraction and LC-MS/MS Analysis 

A total of 18 preserved (at −80 °C) fecal samples (six samples/treatment) were transported to Hangzhou Lianchuan Biotechnology Co., Ltd., Hangzhou, China, by maintaining the cold supply chain. The sample preparation and metabolomics extraction were carried out by following the conventional untargeted metabolomics (LC-MS/MS) protocols detailed on the company site Hangzhou Lianchuan Biotechnology Co., Ltd. Briefly, a 100 mg fecal sample for each ewe was ground in liquid nitrogen; 20 mg fecal samples were mixed with 120 µL of pre-cooled methanol (50%, stored at −20 °C), vortexed for 1 min, incubated at room temperature for 10 min, stored overnight at −20 °C for protein precipitation, centrifuged at 4000 g for 20 min at 4 °C, and supernatants were transferred to 96-well plates. Additionally, a 10 µL extraction mixture for each sample was taken and combined as a pooled quality control (QC) sample. The plates were immediately stored at −80 °C before loading in LC-MS. All samples were acquired by the LC-MS system following machine orders. The chromatographic separations were performed using an ultra-performance liquid chromatography (UPLC) system (SCIEX, Macclesfield, UK). Reversed-phase separation was performed using an ACQUITY UPLC T3 column (100 mm × 2.1 mm, 1.8 µm, Waters, Wilmslow, UK), maintaining a 35 °C column oven temperature with a 0.4 mL/min flow rate. The mobile phase comprised solvent A (water, 0.1% formic acid) and solvent B (phenol, 0.1% formic acid). Gradient elution conditions were 0–0.5 min, 5% B; 0.5~7 min, 5% to 100% B; 7~8 min, 100% B; 8–8.1 min, 100% to 5% B; 8.1–10 min, 5% B. The injection volume for each sample was 4 µL. Eluted metabolites from the column were detected using a high-resolution tandem mass spectrometer TripleTOF5600plus (SCIEX, Macclesfield, UK). The QTOF was configured to operate in both positive and negative ion modes. The curtain gas was set to 30 PSI, the ion source gas1 to 60 PSI, the ion source gas2 to 60 PSI, and the interface heater temperature to 650 °C. The ion spray voltage floating was set to 5000 V and 4500 V for positive and negative ion modes, respectively. The data collection mode was IDA, and TOF’s mass range was adjusted from 60 to 1200 Da. The survey scans were acquired in 150 ms with up to 12 product ion scans over counts per second exceeding 100 at the 1+ charge state. The total cycle time was set at 0.56 s, and a 40 GHz multichannel TDC detector with four anode/channel detectors was monitored by four-time bins summation per scan by adjusting the pulse frequency at 11 kHz. The dynamic exclusion timer was set to 4 s. The mass accuracy was calibrated after every 20 samples during the acquisition. Furthermore, a quality control sample (pool of all samples) was acquired after every 10 samples to evaluate the stability of the LC-MS throughout the acquisition.

### 2.3. Bioinformatic Analysis

The peak picking, peak grouping, retention time correction, second peak grouping, and isotope and adduct annotation of the MS data file were processed using XCMS software. The raw data containing LC-MS files were converted to mzXML format using the R packages including XCMS (Online, version 3.4.4), CAMERA (version 1.38.1), and MetaX (version 2.29). The ionic identification was carried out by combining the retention time (RT) and m/z values. The three-dimensional matrix was generated with arbitrarily assigned peak indices (retention time *m*/*z* value of pairs), sample names (observations), and ion intensity information (variables). The metabolites were annotated using the online KEGG (http://www.genome.jp/kegg/, 29 July 2023) and HMDB databases (https://hmdb.ca/, 29 July 2023) by matching the exact molecular mass data (*m*/*z*) of samples with those from the database. The metabolites were annotated by adjusting the mass difference between the observed and database values <10 ppm, their molecular formula was identified, and they were validated using isotopic distribution measurements. Additionally, an in-house fragment spectrum library was used to validate the metabolite identification. MetaX was used to further preprocess the peak data’s intensity. Those features found in less than 50% of QC samples or 80% of biological samples were removed, and the remaining peaks with missing values were imputed using the k-nearest-neighbor algorithm to improve data quality. The log-transformed (base 10) data were scaled using the central mean divided by the standard deviation of the variables. The normalized data were subjected to principal component analysis (PCA) and orthogonal projection to latent structures discriminant analysis (OPLS-DA) to compare the differences among different metabolites using MetaboAnalyst 5.0 (https://www.metaboanalyst.ca/, 29 July 2023). Metabolite screening was carried out by variable importance in projection (VIP) scores of the OPLS-DA model and statistical significance as per Student’s t-tests. Using the preprocessed dataset, PCA was used to detect outliers and evaluate batch effects. To minimize signal intensity drift over time, a robust LOESS signal correction based on quality control was fitted to the QC data concerning the order of injection. Furthermore, the relative standard deviations of the metabolic features were calculated across all QC samples, and those greater than 30% were eliminated.

## 3. Results

### 3.1. Identification and Quantification of Metabolites in the Fecal Samples

To evaluate the metabolomic changes induced by dietary protein levels, the untargeted screening of fecal samples of lactating ewes was performed using a high-resolution mass spectrometer. In the current study, a total of 10,243 metabolites were identified, and 6660 were annotated in positive ionic mode, while 4456 were in negative ionic mode. By using MetaboAnalyst, 544 and 325 metabolites were identified according to level 2 of confidence in positive and negative ionic modes, respectively.

### 3.2. Fecal Metabolomic Profiles 

**Figure 1:**
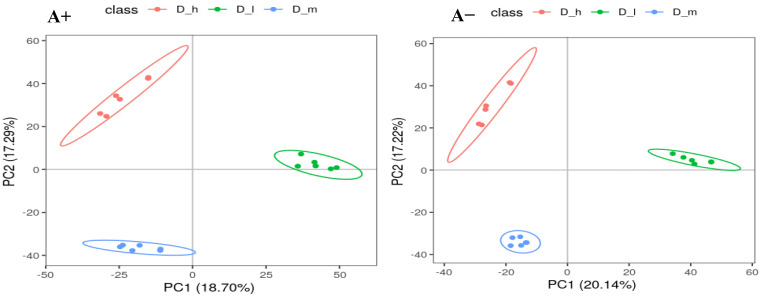
PCA analysis of positive (A+) and negative (A−) ionic modes detected fecal metabolites of the lactating ewes fed different dietary protein levels (D_h = high, D_m = medium, and D_I = low dietary protein levels).

**Figure 2:**
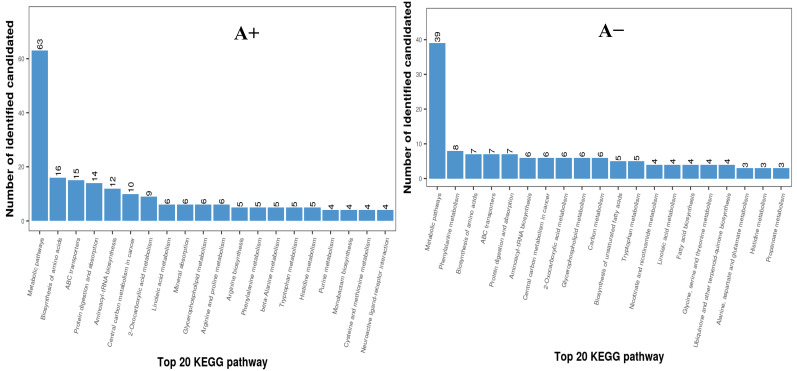
Enriched chemical class KEGG pathways of positive (A+) and negative (A−) ionic mode detected fecal metabolites of the lactating ewes fed different dietary protein levels (D_h = high, D_m = medium, and D_I = low dietary protein levels).

The PCA analysis and KEGG pathway enrichment provided an overall picture of variations in metabolite profile caused by feeding with varying dietary protein levels. The number of samples is represented by the dots in the PCA plot, and their aggregation or separation patterns show their similarity or differences (Figure 1). The PCA analysis revealed a distinct separation for data from different treatments, indicating that dietary protein levels had a substantial influence on the metabolomic profile of nursing sheep. The degree of correlation in metabolite abundance was used to determine the changes in fecal metabolites of lactating ewes produced by feeding with varied protein levels. The heatmap dendrogram (Supplementary Figure S1a,b) revealed that D_h-induced metabolites were tightly clustered across the groups. Potential biomarkers were found to contribute to several processes, including fatty-acid metabolism, bile-acid production, purine, and protein metabolism, according to the KEGG pathway enrichment analysis (Figure 2). As a consequence, the enrichment of pathways involved in metabolism and biochemical synthesis may suggest that variations in dietary protein levels affect metabolism, and ultimately the performance of the lactating ewes (Figure 2).

### 3.3. Metabolic Pathways of Differential Metabolites

**Figure 3:**
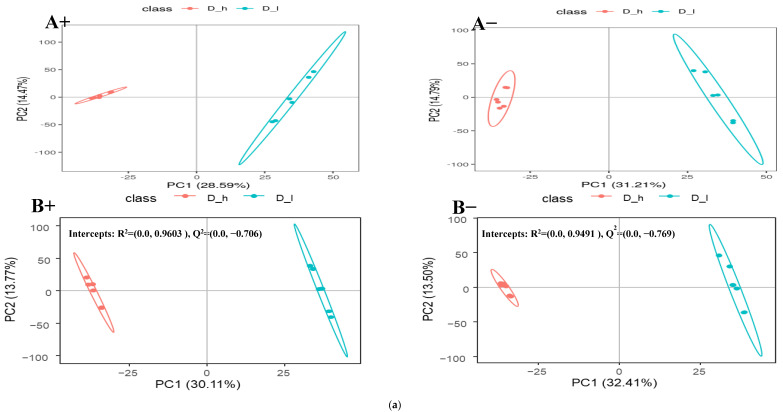

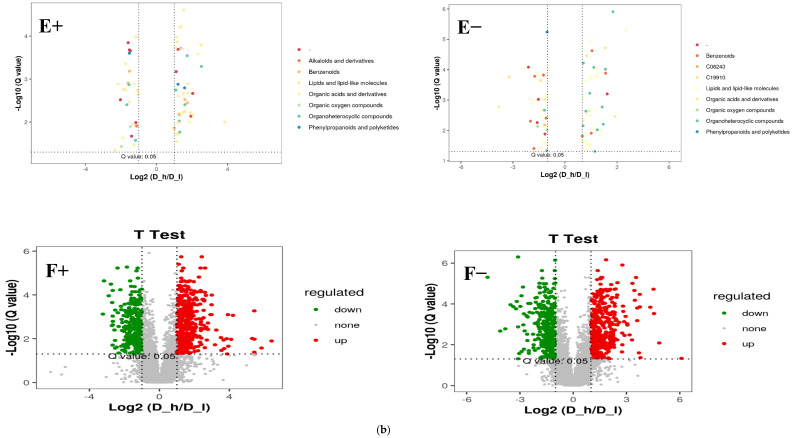
(**a**) Comparative PCA and PLS-DA analysis of positive (A+ and B+) and negative (A− and B−) ionic mode detected fecal metabolites of the lactating ewes fed D_h = high and D_I = low dietary protein levels; (**b**) comparative volcano plots of positive (E+ and F+) and negative (E− and F−) ionic mode detected fecal metabolites of the lactating ewes fed D_h = high and D_I = low dietary protein levels.

**Figure 4:**
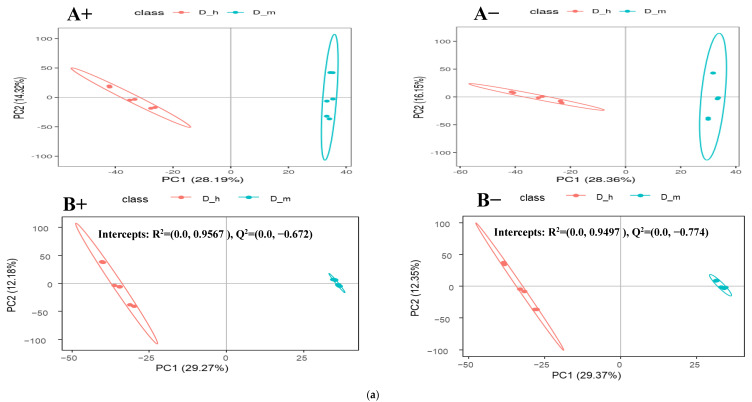

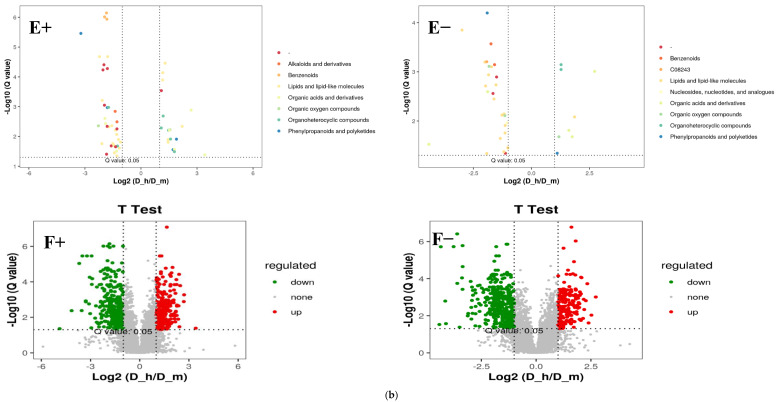
(**a**) Comparative PCA and PLS-DA analysis of positive (A+ and B+) and negative (A− and B−) ionic mode detected fecal metabolites of the lactating ewes fed D_h = high and D_m = medium dietary protein levels. (**b**) Comparative volcano plots of positive (E+ and F+) and negative (E− and F−) ionic mode detected fecal metabolites of the lactating ewes fed D_h = high and D_m = medium dietary protein levels.

**Figure 5:**
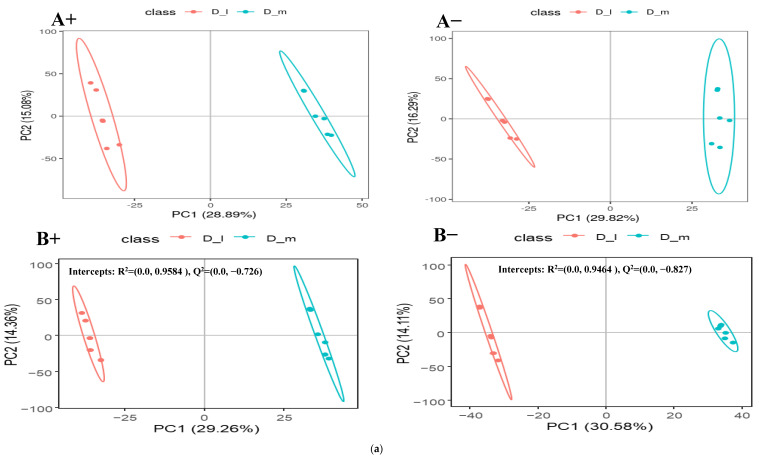

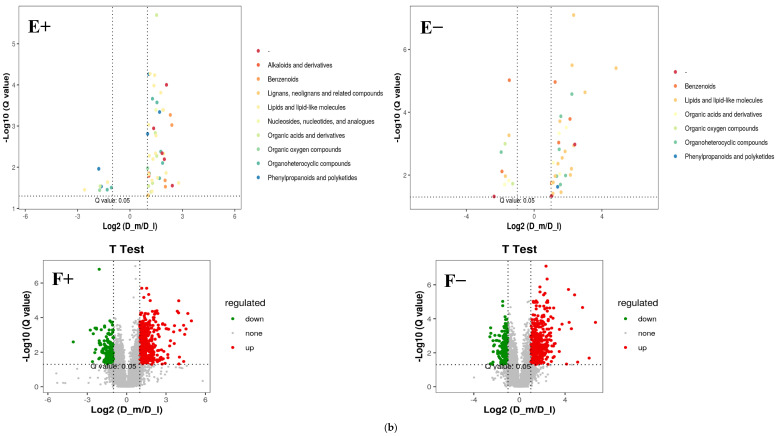
(**a**) Comparative PCA and PLS-DA analysis of positive (A+ and B+) and negative (A− and B−) ionic mode detected fecal metabolites of the lactating ewes fed D_m = medium and D_I = low dietary protein levels. (**b**) Comparative volcano plots of positive (E+ and F+) and negative (E− and F−) ionic mode detected fecal metabolites of the lactating ewes fed D_m = medium and D_I = low dietary protein levels.

The differential metabolites among these dietary groups were screened out by adjusting the statistical standards at q-value < 0.05, VIP score > 1, and ratio > 2 which are summarized in volcano plots for both positive and negative ionic modes (Figure 3b, Figure 4b and Figure 5b). Based on the PLS-DA analysis, discriminated metabolites that contributed to the metabolomic difference in pairwise comparisons were marked with their VIP scores, coefficient variation, regulation type (up or down), and statistical differences (*p*-value) for both positive and negative ionic modes (Table 3 and Table 4). The pairwise comparison (D_h/D_m, D_h/D_I, and D_m/D_I) showed significant differences for the fecal metabolites’ concentrations of the four superclasses (benzenoids, lipids and lipid-like molecules, organic acids and their derivatives, and organoheterocyclic compounds) and five superclasses (benzenoids, lipids and lipid-like molecules, organic acids and derivatives, organic oxygen compounds, and organoheterocyclic compounds). In a positive mode, four metabolites of the benzenoids, nineteen of the lipids and lipid-like molecules, nine of the organic acids and derivatives, and eight of the organoheterocyclic compounds had significant differences across the pairwise comparison (Table 3). Similarly, five of the benzenoid-based, twenty-five of the lipid- and lipid-like-molecule-based, eight of the organic-acid-and-derivative-based, two of the organic-oxygen-compound-based, and four of the organoheterocyclic-compound-based metabolites were significantly influenced by the dietary protein levels in the negative ionic mode (Table 4). The detected differential metabolites in the current study were mostly carbohydrates, amino acids, and their derivatives, lipids, purine, and cofactors. The partial least-squares discriminant analysis (PLS-DA) is a powerful method that classifies the samples through the discrimination of ion peaks and removing noncorrelated variations within spectra. Therefore, a comparative PLS-DA was carried out to find the potential biomarkers between D_h-, D_m-, and D_I-fed groups. The PLS-DA plots showed clear comparative separation between the D_h/D_m, D_h/D_I, and D_m/D_I groups (B+ and B- sections of Figure 3a, Figure 4a and Figure 5a). Based on the first two components, the PLS-DA plot showed that D_h had better separation than D_m and D_I, respectively. The intercepts for the comparative pairs (D_h/D_m, D_h/D_I, and D_m/D_I) in the positive as well as negative ionic modes are represented with R2 and Q2, which indicate the goodness of fit and prediction ability of the model, respectively. Similarly, pairwise PCA analysis reveals a higher degree of aggregation within the D-h group than all other pairs. In both the PCA and PLS-DA plots, the comparative separation among the D_h/D_m, D_h/D_I, and D_m/D_I groups was superior in positive as well as negative ionic modes, which indicated that sheep fed higher protein levels had alterations in the levels of the metabolites.

## 4. Discussion

The rumen is the main organ that determines the digestion efficiency of ruminants. Diet-associated factors such as feed ratio, dietary ingredient sources, and level of nutrients can influence the rumen ecology by altering the microbial composition and level of metabolites. The feces result from GIT metabolism; therefore, its associated parameters such as consistency, nutrient composition, and metabolite profiles can be used as biomarkers to investigate rumen health and metabolic pathways. Dietary protein is a major nutrient that provides amino acids for several metabolic pathways involved in tissue synthesis, regulation of metabolic processes, and immune as well as redox strengthening, which ultimately improves the growth performance of the ruminants [19,20]. In our recently published work, we evaluated how dietary protein levels (same diets as used in this experiment) change the fecal bacterial composition and found that changes in dietary protein contents altered rumen microbiota composition [17] and consequently influenced the growth performance, milk production, and blood biochemical indices [18] of the nursing ewes. Further, it is well established that dietary protein levels influence growth performance by altering the microbial and enzymatic function of sheep and cattle [5,21]. In the current experiment, the composition of the fecal metabolites significantly differed among groups based on PLS-DA analysis, suggesting that the dietary protein levels alter the rumen metabolomic profiles of lactating ewes. Our results are consistent with [5,22] as they reported significant alterations in rumen metabolomic composition because of dietary protein levels and feed type in Tibetan sheep and yaks, respectively. These results were comparable with the previous study carried out by [22], who found that feed type could significantly change the metabolites and functional pathway of yaks. In this experiment, more than 40 metabolites in both positive as well as negative ionic modes differed significantly across the treatment groups, and notably, most of these biomarkers were increased by feeding sheep increasing dietary protein levels. In our study, trend lines between the groups revealed that organic acids and their associated metabolites are the most significantly affected metabolites detected in the fecal samples. Our results are in line with the previous study [23] as they also documented organic acids’ most altering metabolites in the rumen samples of the yaks fed different dietary protein levels. The possible reason might be more protein availability for degradation, which results in greater amino-acid availability in the small intestine for microbial downstream to supply the end products for liver metabolism. However, unabsorbed amino acids can be fermented by hindgut microbes to produce sugar intermediate products including organic acids [24]. Therefore, greater variation in the concentration of the organic-acid-based metabolites in the present experiment suggests that organic-acid metabolism is highly sensitive to changes in the dietary protein contents. In our study, aspartic acid, phenylethylamine, tyrosine, tryptophan, and methionine sulfoxide showed significant differences between the different groups, and notably, all these metabolites showed a clear increasing trend with increasing dietary protein levels. The difference between groups can be associated with the differences in dietary protein levels as it is well known that dietary protein contents provide nitrogen bases as well as amino acids for metabolism [17]. Regarding the amino-acid compounds, it is established that there are correlations with the dietary protein levels as followed by the nursing ewes in the current experiment. For example, aspartic acid is a non-essential amino acid that is involved in protein synthesis and several metabolic pathways [25]. Dietary protein also provides aspartic acid for nitrogen metabolism in ruminants [26], so it can be assumed that dietary protein could have a direct influence on both essential as well as non-essential amino-acid profiles. However, how much dietary protein influences the aspartic-acid levels in the animal’s tissues is complex as various factors, including the animal’s metabolic rate, protein synthesis rates, and overall amino-acid balance, can influence the actual concentration of aspartic acid in the tissues [9,27]. Phenylethylamine is an essential amino acid that is involved in energy metabolism (glucose and fat) and synthesis of certain types of hormones as well as neurotransmitters [28]. The metabolism of tryptophan is a complex multi-pathway process that involves the host’s intestinal microbiota through symbiotic relationships [29]. The tryptophan metabolism generates several metabolites as a coproduct of these pathways, and these coproducts strengthen the immune function of the animal [30]. Research has evidenced that tryptophan metabolism by the host’s intestinal microorganisms produces key tryptophan metabolites including anabolic D-tryptophan (synthesized by symbiotic bacteria), small peptides (synthesized by fungi), and indoles and their derivatives (synthesized by bacteria and protozoa) [31]. Indoleacetic acid, a coproduct of the tryptophan metabolism, was increased in the higher-protein-fed group in this experiment, which supports the above points. It is established that animal intestinal bacteria metabolized the tryptophan into different indole derivatives including tryptamine, indoleacetic acid, indole sulfuric acid, and indole-3-acetaldehyde [32]. The ruminal microbes might hydrolyze tyrosine into L-DOPA (l-3,4-dihydroxyphenylalanine) by tyrosine hydroxylase, which allows ruminal bacteria to use nitrogen for microbial protein. One study [33] reported increased L-DOPA concentrations when feeding with those feeds containing higher degradable components contents, explaining the increased metabolism of tyrosine in higher-protein-level-fed sheep in the current study. Methionine is the first limiting amino acid for ruminants, meaning they must obtain it from their diet to cope with production and reproduction efficiency [34]. Methionine sulfoxide is an oxidized form of methionine that has key responsibilities including maintaining the proper level of methionine by preventing excessive oxidation of methionine and various metabolic processes in ruminants [35]. However, understanding of ruminants’ metabolites is still far behind that of other animals [36], and even some detected metabolites in this study have not been reported before and require more insights. In our study, the enrichment of indoleacetic acid, lucidenic acid, corosolic acid, hydroxy icosanoic acid, and mevalonic acid was increased by feeding the higher dietary protein level. These compounds have diverse functions in biological systems, but their roles in ruminant metabolism are not extensively documented. Corosolic acid has been investigated for potential effects on glucose metabolism and insulin sensitivity in animals [37]. Its role in ruminant metabolism is not widely studied, but it is possible that similar effects on glucose metabolism could be relevant for ruminants as well. Mevalonic acid is a key precursor in the biosynthesis of various compounds, including sterols (such as cholesterol) and isoprenoids (which include compounds like hormones, vitamins, and carotenoids) [38]. It is important to note that ruminants do not synthesize cholesterol in the same way that non-ruminants do [39], and their synthesis of other compounds could be influenced by their unique digestive and metabolic processes. Based on these results, we speculate that a higher supply of dietary protein may improve the metabolomics and growth performance in sheep by contributing to nutrient absorption. Metabolomics provides insights into changes in metabolites by external intervention and provides the true picture of the physiochemical status of the animals [40,41]. Compared to microorganisms, the metabolome can reflect the most intuitive physiological state of the animal [41]. Therefore, the metabolomic profiles of fecal samples in the current study were sensitive to reflect how dietary protein levels changed the ruminal metabolomic profile. The results of the current study project that the ruminal microflora, particularly bacterial groups, may be involved in the positive regulation of dietary amino acids (aspartic acid, phenylethylamine, tyrosine, tryptophan, and methionine sulfoxide), as well as organic acids (indoleacetic acid, lucidenic acid, corosolic acid, hydroxy icosanoic acid, and mevalonic acid), and ultimately promoted growth performance in sheep. In terms of pathway enrichment, the dietary protein had the highest impact on the metabolic pathways (63 metabolites), followed by the biosynthesis of amino acids (16 metabolites) and the ABC transporters (15 metabolites) in this study. The understanding of dietary protein influences on metabolic pathways is complex as it depends upon several factors including the specific amino-acid composition of the diet, the animal’s physiological state, and its overall nutritional balance [42]. Amino acids from dietary protein and microbial protein contribute to protein synthesis in the animal’s body [9]. It is established that rumen bacteria are directly associated with diet, and dietary regimen changes can significantly affect the body’s amino-acid and digesta composition [43]. Zhang et al. [44] reported that feeding diets varying in dietary constituents resulted in alterations in the metabolic pattern as well as amino-acid profile in ruminal metabolites of dairy cows. Another study by Wang et al. [45] discovered that rumen bacteria (particularly Schwartzia and Moryella species) are involved in amino-acid profiling and tissue deposition in Tan sheep. This includes the synthesis of structural proteins, enzymes, and other functional proteins. Lactating sheep require a sufficient dietary supply of protein to support milk production [9].

## 5. Conclusions

In summary, this study correlated fecal metabolomics and the association between different metabolites with dietary protein concentrations. It enhanced our basic understanding of ruminal metabolites and provided knowledge guidelines in this field and the protein requirements of lactating ewes. Further, it provided more insights regarding how dietary protein levels influenced the concentrations of different metabolites particularly involved in protein synthesis and energy metabolic pathways and more detail about their mechanisms of action in rumen metabolism.

## Figures and Tables

**Table 1:** The feed formulation is on a dry basis.

Ingredients	Diets ^1^		
	D_l	D_m	D_h
Corn grains	28.2	26.0	19.05
Soybean meal	5.4	8.65	18.6
Corn starch	8.65	7.6	4.7
Corn silage	40.0	35.0	34.0
Wheat straw	13.0	10.0	10.0
Bean powder	2.0	10.0	11.0
Calcium carbonate	0.55	0.6	0.65
Calcium hydrogen phosphate	0.55	0.5	0.35
Salt	0.3	0.3	0.3
Baking soda	0.35	0.35	0.35
Premix	1.0	1.0	1.0
Total	100.0	100	100.0

^1^ Diets = D_I, D_m, and D_h, which are low-protein (8.58% on a dry basis), medium-protein (10.34% on a dry basis), and high-protein (13.93% on a dry basis) diets.

**Table 2:** The feed chemical composition is on a dry basis.

Nutrients	Diets ^1^		
	D_l	D_m	D_h
Metabolizable energy (MJ/kg)	9.45	9.47	9.47
Crude protein (%)	8.58	10.34	13.93
Neutral detergent fiber (%)	32.17	32.01	32.52
Acid detergent fiber (%)	17.24	17.71	18.44
Calcium (%)	0.69	0.71	0.71
Phosphorus (%)	0.38	0.39	0.39

^1^ Diets = D_I, D_m, and D_h, which are low-protein (8.58% on a dry basis), medium-protein (10.34% on a dry basis), and high-protein (13.93% on a dry basis) diets.

**Table 3:** Pairwise comparison of the discriminated metabolites changed by dietary treatments (positive ionic mode).

Superclass	Metabolites	Pairwise Comparison											
		D_h and D_I				D_h and D_m				D_I and D_m			
		VIP	CV	*p*-Value	RG	VIP	CV	*p*-Value	RG	VIP	CV	*p*-Value	RG
Benzenoids	2-methoxy-4-pentadecylbenzoic acid	2.11	0.14	0.01	Up	-	-	-	-	1.82	0.14	0.01	Up
	2-Phenylethanaminium	2.10	0.01	0.00	Up	-	-	-	-	3.02	0.01	0.00	Up
	C.I. Pigment Red 149	2.13	0.05	0.00	Down	-	-	-	-	-	-	-	-
	Styrene	2.25	0.03	0.00	Up	-	-	-	-	3.08	0.03	0.00	Up
Lipids and lipid-like molecules	(22*E*,24*R*)-Stigmasta-4,22-diene-3,6-dione	2.49	0.04	0.01	Up	1.79	0.04	0.00	Up	-	-	-	-
	11-Hydroxyeicosatetraenoate glyceryl ester	2.35	0.06	0.00	Up	-	-	-	-	2.20	0.06	0.00	Up
	12-Ketodeoxycholic acid	2.22	0.29	0.01	Up	-	-	-	-	1.84	0.05	0.01	Up
	1-Heptadecanoyl-sn-glycero-3-phosphocholine	1.83	0.07	0.03	Down	1.71	0.07	0.02	Down	-	-	-	-
	2-Linoleoylglycerol	1.65	0.11	0.00	Down	2.07	0.11	0.04	Down	-	-	-	-
	7-Oxostigmasterol	2.59	0.10	0.00	Up	2.18	0.03	0.01	Down	1.96	0.10	0.01	Up
	8*Z*,14*Z*-Eicosadienoic acid	2.01	0.36	0.01	Up	-	-	-	-	-	-	-	-
	9,10-Dihydroxy-12*Z*-octadecenoic acid	-	-	-	-	3.10	0.07	0.00	Up	1.84	0.07	0.02	Down
	Camellenodiol	2.66	0.06	0.00	Up	1.93	0.06	0.00	Up	-	-	-	-
	Cortisone	1.69	0.30	0.03	Up	-	-	-	-	2.19	0.30	0.00	Up
	Hydrocortisone	1.56	0.04	0.00	Down	3.10	0.04	0.00	Down	1.46	0.04	0.00	Up
	Isopropyl tiglate	2.41	0.03	0.05	Down	-	-	-	-	2.67	0.03	0.04	Down
	Linoleic acid methyl ester	2.64	0.10	0.00	Down	2.50	0.10	0.02	Down	-	-	-	-
	LysoPE 15:0	2.18	0.28	0.00	Down	2.11	0.28	0.02	Down	-	-	-	-
	LysoPE 16:0	2.28	0.04	0.00	Down	2.80	0.04	0.00	Down	-	-	-	-
	LysoPE 18:0	2.60	0.13	0.00	Down	1.65	0.13	0.02	Down	-	-	-	-
	Oleyl alcohol	1.89	0.05	0.01	Down	2.51	0.05	0.00	Down	-	-	-	-
	Traumatin	2.60	0.07	0.01	Up	-	-	-	-	2.62	0.07	0.00	Up
	Zymosterol	2.07	0.08	0.00	Up	1.85	0.17	0.00	Up	-	-	-	-
Organic acids and derivatives	Aspartic acid	2.70	0.10	0.00	Up	3.29	0.10	0.00	Up	-	-	-	-
	Methionine sulfoxide	2.30	0.17	0.00	Up	-	-	-	-	-	-	-	-
	*N*-Acetyl-dl-valine	1.94	0.16	0.00	Up	-	-	-	-	2.31	0.16	0.00	Up
	*N*-Acetyl-d-norleucine	2.01	0.07	0.02	Up	-	-	-	-	1.81	0.07	0.03	Up
	Proline	-	-	-	-	-	-	-	-	1.38	0.16	0.04	Up
	Tiglylglycine	2.67	0.08	0.01	Up					1.88	0.08	0.02	Up
	Tyrosine	-	-	-	-	2.42	0.07	0.03	Up	2.42	0.07	0.03	Down
	2,2,6,7-Tetramethylbicyclo[4.3.0]nona-1(9),4-diene-7,8-diol	-	-	-	-	-	-	-	-	2.40	0.24	0.04	Down
	Trehalose	1.93	0.34	0.01	Down	3.09	0.34	0.00	Down	-	-	-	-
Organoheterocyclic compounds	Tryptophan	2.22	0.07	0.02	Up	-	-	-	-	-	-	-	-
	Indole-3-pyruvic acid	2.28	0.07	0.00	Up	-	-	-	-	2.43	0.07	0.00	Up
	Indoleacetic acid	3.04	0.07	0.00	Up	-	-	-	-	-	-	-	-
	Isonicotinic acid	2.21	0.22	0.00	Up	-	-	-	-	2.08	0.22	0.00	Up
	Methylimidazoleacetic acid	2.53	0.18	0.00	Up	-	-	-	-	2.27	0.18	0.00	Up
	Pergolide	2.38	0.08	0.00	Down	-	-	-	-	-	-	-	-
	Phaeophorbide b	2.24	0.20	0.00	Down	2.54	0.20	0.00	Down	-	-	-	-
	Protoporphyrin IX	1.92	0.14	0.03	Down	2.13	0.14	0.02	Down	-	-	-	-

**Table 4:** Pairwise comparison of the discriminated metabolites changed by dietary treatments (negative ionic mode).

Superclass	Metabolite	Pairwise Comparison											
		D_h and D_I				D_h and D_m				D_I and D_m			
		VIP	CV	*p*-Value	RG	VIP	CV	*p*-Value	RG	VIP	CV	*p*-Value	RG
Benzenoids	3-Ethylphenol	-	-	-	-	-	-	-	-	2.10	0.13	0.01	Down
	3-Methylphenylacetic acid	-	-	-	-	-	-	-	-	1.93	0.11	0.00	Up
	4-Ethyl-2-methoxyphenol	-	-	-	-	-	-	-	-	1.86	0.05	0.02	Up
	4-Methylhippuric acid	-	-	-	-	-	-	-	-	2.56	0.34	0.00	Up
	D8’-Merulinic acid A	-	-	-	-	-	-	-	-	2.04	0.11	0.00	Up
Lipids and lipid-like molecules	12-hydroxyheptadecanoic acid	1.96	0.41	0.03	Up					2.72	0.04	0.01	Up
	2(*R*)-hydroxy icosanoic acid	2.52	0.11	0.00	Up	-	-	-	-	-	-	-	-
	2-Hydroxy-2-methylbutyric acid	1.82	0.35	0.00	Down	-	-	-	-	2.46	0.35	0.01	Down
	2-Hydroxymyristic acid	1.58	0.03	0.00	Up	-	-	-	-	2.39	0.03	0.00	Up
	3a,7b,12a-Trihydroxyoxocholanyl-Glycine	1.74	0.03	0.03	Down	1.81	0.03	0.04	Down	-	-	-	-
	Corosolic acid	2.21	0.08	0.00	Up	-	-	-	-	-	-	-	-
	Dodecanedioic acid	2.59	0.07	0.01	Up					2.76	0.07	0.01	Up
	Furfuryl isovalerate	1.63	0.11	0.01	Up	-	-	-	-	-	-	-	-
	Heptadecanoic acid	-	-	-	-	-	-	-	-	2.02	0.13	0.01	Up
	Lucidenic acid L	1.83	0.20	0.03	Up	-	-	-	-	-	-	-	-
	Lucidenic acid N	1.57	0.01	0.00	Up	2.84	0.01	0.00	Down	4.32	0.01	0.00	Up
	LysoPC 18:0	1.68	0.20	0.00	Down	2.08	0.20	0.02	Down	-	-	-	-
	LysoPC 18:1	1.75	0.38	0.01	Down	-	-	-	-	-	-	-	-
	LysoPE 14:0	1.52	0.31	0.02	Down	2.72	0.31	0.00	Down	-	-	-	-
	LysoPE 15:0	2.62	0.05	0.00	Down	2.28	0.05	0.00	Down	-	-	-	-
	LysoPE 16:0	2.10	0.05	0.00	Down	2.79	0.05	0.00	Down	-	-	-	-
	LysoPE 18:0	2.64	0.10	0.00	Down	2.19	0.10	0.00	Down	-	-	-	-
	LysoPE 18:1	2.28	0.02	0.00	Down	1.87	0.02	0.01	Down	-	-	-	-
	LysoPG 14:0; LysoPG 14:0	1.65	0.07	0.04	Down	2.27	0.07	0.05	Down	-	-	-	-
	LysoPG 15:0; LysoPG 15:0	1.45	0.32	0.02	Up	1.98	0.06	0.02	Down	2.29	0.32	0.00	Up
	LysoPS 16:0	1.72	0.14	0.01	Down	2.76	0.14	0.00	Down	-	-	-	-
	Mevalonic acid	2.00	0.04	0.00	Up	-	-	-	-	-	-	-	-
	Myristic acid	1.84	0.06	0.01	Up					2.86	0.06	0.00	Up
	Palmitic acid	-	-	-	-	-	-	-	-	1.86	0.04	0.02	Up
	Polyethylene, oxidized	2.12	0.11	0.00	Up	-	-	-	-	-	-	-	-
	Ursocholic acid	2.37	0.02	0.01	Up	-	-	-	-	-	-	-	-
Organic acids and derivatives	2-Hydroxy-2-(2-oxopropyl)butanedioic acid	3.18	0.04	0.00	Down	3.49	0.04	0.03	Down	-	-	-	-
	d-(+)-Pantothenic acid	2.01	0.04	0.00	Down	-	-	-	-	-	-	-	-
	l-Aspartic acid	3.13	0.07	0.00	Up	3.07	0.07	0.00	Up	-	-	-	-
	l-Tyrosine	2.27	0.26	0.02	Up	2.27	0.26	0.02	Up	2.08	0.26	0.02	Down
	*N*-Acetyl-d-norleucine	2.12	0.00	0.00	Up	-	-	-	-	1.82	0.00	0.00	Up
	*N*-Acetyl-l-phenylalanine	1.67	0.02	0.02	Up	-	-	-	-	1.85	0.02	0.02	Up
	*N*-Palmitoyl phenylalanine	1.43	0.06	0.00	Up	-	-	-	-	2.66	0.06	0.00	Up
	*N*-Phenylacetylglutamic acid	-	-	-	-	-	-	-	-	1.89	0.12	0.00	Up
Organic oxygen compounds	1,5-Anhydro-d-sorbitol	2.19	0.16	0.01	Down	-	-	-	-	2.35	0.16	0.00	Down
	Maltotriose	1.49	0.24	0.01	Down	2.76	0.24	0.00	Down	-	-	-	-
Organoheterocyclic compounds	Kynurenic acid	2.15	0.17	0.00	Up	2.09	0.17	0.00	Up	-	-	-	-
	Paramethadione	2.83	0.11	0.01	Up	-	-	-	-	2.07	0.11	0.02	Up
	Tryptophan	2.04	0.05	0.00	Up	-	-	-	-	-	-	-	-
	Xanthine	2.49	0.05	0.05	Up	-	-	-	-	-	-	-	-
Phenylpropanoids and polyketides	Hydroxygaleon	-	-	-	-	-	-	-	-	2.05	0.26	0.02	Up

## Data Availability

Data are contained within the article and supplementary materials.

## Supplementary Materials

The following supporting information can be downloaded at: https://www.mdpi.com/article/10.3390/ani14010121/s1, Figure S1: (a) Heatmap analysis of fecal metabolites of lactating ewes fed different levels (D_h, D_I, and D_m) of protein (positive ionic mode); (b) heatmap analysis of fecal metabolites of lactating ewes fed different levels (D_h, D_I, and D_m) of protein (negative ionic mode).
