# Supplementary material for: Effects of Dietary Protein Levels on Sheep Gut Metabolite Profiles during the Lactating Stage
Source: Animals (Basel). 2023 Dec 29;14(1):121. doi: 10.3390/ani14010121 (PMC10778572; doi:10.3390/ani14010121)
Supplement: Supplementary file 1 [file animals-14-00121-s001.zip › animals-2749261-supplementary.pdf]

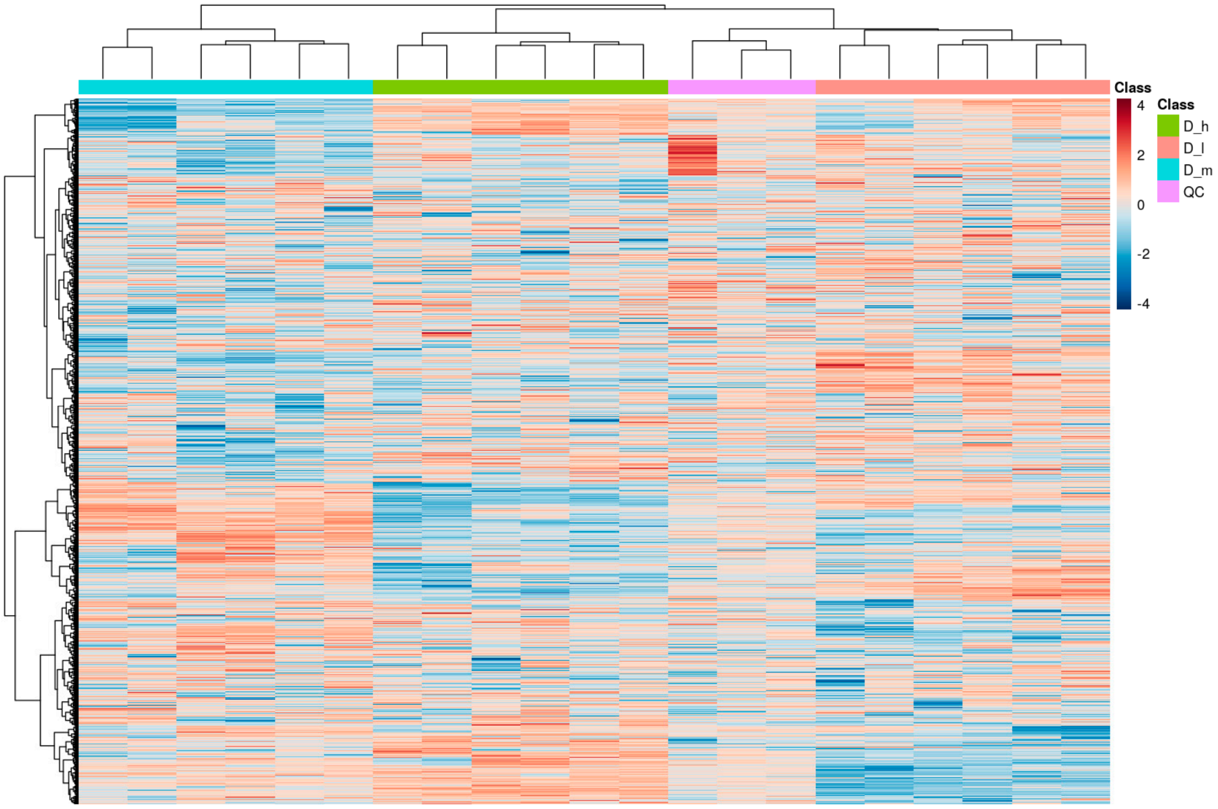

Figure S1a. Heat Map Analysis of Fecal Metabolite of Lactating Ewes fed different levels (D_h, D_I, and D_m) of protein (Positive Ionic Mode)

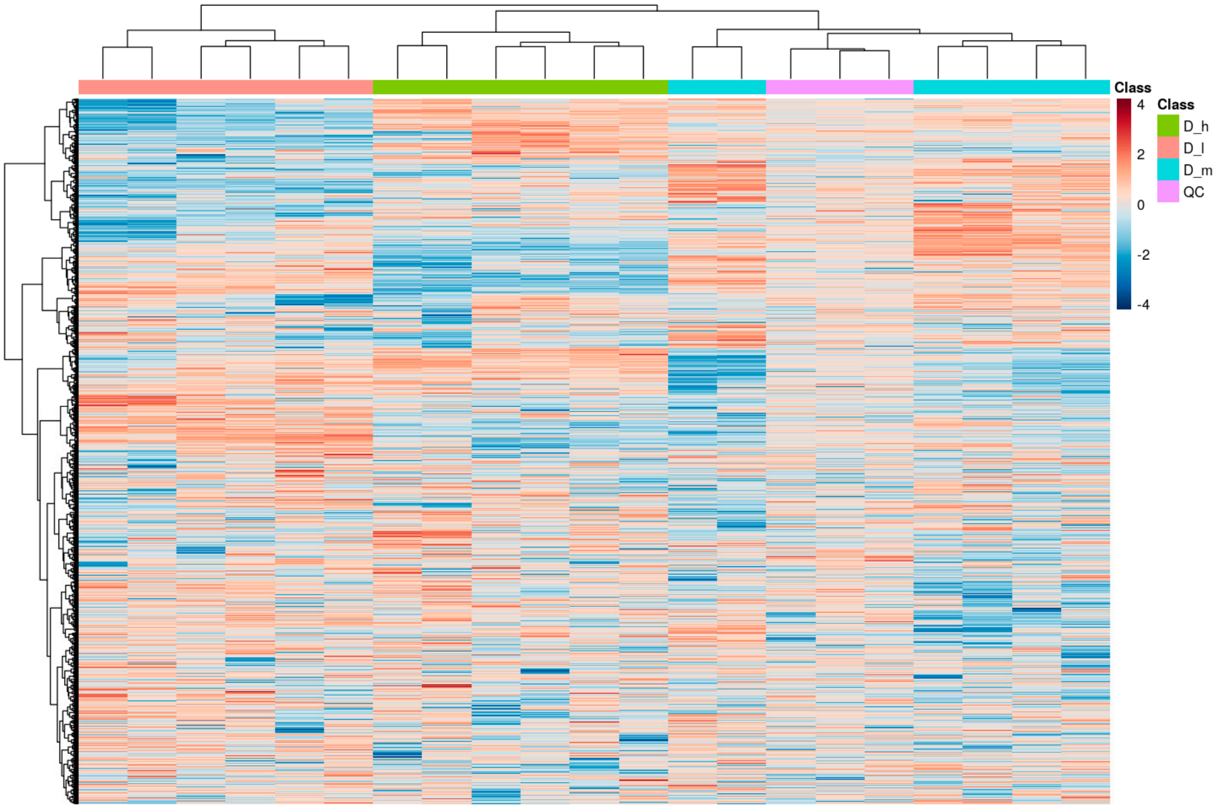

Figure S1b. Heat Map Analysis of Fecal Metabolite of Lactating Ewes fed different levels (D_h, D_I, and D_m) of protein (Negative Ionic Mode)
